# Supplementary material for: ‘Can you please hold my hand too, not only my breast?’ The experiences of Muslim women from Turkish and Moroccan descent giving birth in maternity wards in Belgium
Source: PLoS One. 2020 Jul 29;15(7):e0236008. doi: 10.1371/journal.pone.0236008 (PMC7390407; doi:10.1371/journal.pone.0236008)
Supplement: S1 Appendix — (DOCX) [file pone.0236008.s001.docx]

**Additional file 1: Examples of interview questions**

**Introduction**

Can you tell me something about the family in which you grew up?

Can you tell me about your family now?

**Opening Questions**

Can you describe what happened during your pregnancy?

- Can you tell me about the check-ups?
- Can you tell me about the contact with the gynaecologist/ midwife?
- Can you describe what happened before your delivery?

What did you expect of giving birth before you went to the hospital?

**Leading Questions**

Can you tell me all about your delivery?

- Who took care of you?
- What happened next?
- Can you tell me how you felt when x happened?
- Can you describe how caregivers reacted to x?
- Can you describe how you felt when caregivers reacted this way?

What happened directly after the delivery?

- What did the gynaecologist/nurses do after the delivery?
- Can you describe what happened when they took care of you?
- Can you tell me about the care for the baby?
- Can you tell me how you felt when x happened?
- Can you describe how caregivers reacted to x?

Can you describe what happened after your delivery?

- Can you describe what happened in the hospital in the first days with your new-born?
- Can you describe the events that happened in the morning/ at night?
- What happened when gynaecologist/midwives came to check you?
- What happened when the paediatricians/midwives came to help you with the baby?
- Can you tell me how you felt when x happened?
- Can you describe how caregivers reacted to x?
- Can you describe how you felt when caregivers reacted this way?

Can you tell me about rituals or traditions surrounding the birth that you carried out in the hospital?

- Can you describe how caregivers reacted to x?
- Can you describe how you felt when caregivers reacted this way?

Can you describe what happened when the caregivers discharged from the hospital?

- Can you describe how you felt when leaving the hospital?

Can you describe how you think now about your stay in the hospital?

Can you describe now, in the ideal situation what good care would mean to you?

Can you describe now, what poor care would mean to you?

- Can you tell me in which circumstances you would feel bad about care?

**Concluding Questions**

Now, if you look back at your delivery, are there things left you would like to say to your gynaecologist or nurses or other caregivers?

Many things were discussed, but what matters the most to you?

Are there things that were important to you that we did not discussed yet?
