# Supplementary material for: ‘Can you please hold my hand too, not only my breast?’ The experiences of Muslim women from Turkish and Moroccan descent giving birth in maternity wards in Belgium
Source: PLoS One. 2020 Jul 29;15(7):e0236008. doi: 10.1371/journal.pone.0236008 (PMC7390407; doi:10.1371/journal.pone.0236008)
Supplement: S2 Appendix — (DOCX) [file pone.0236008.s002.docx]

**Additional file 2: Ethical considerations throughout the interview process**

| **Ethical considerations** |
| --- |
| - **Trust and repeated contact with participating women** - Regular meetings face to face or by phone happened prior to the interview to introduce and to get familiar with the interviewer - Preferable time, place and language were carefully checked |
| - **Being properly informed** - Time was given to (re)think the women’s participation by sending the information brochures prior to the interview - The interviewer checked again after some time if the women were still willing to participate - To ensure that the women were properly informed, oral explanation of the full information brochure was necessary - Additional questions were clarified in an easy pace |
| - **Confidentiality & Consent** - Written informed consent was obtained together with some demographic information - Agreement was asked for digitally recording - Identifiable information was treated with confidentiality and only the research team had access to the recordings and interview transcripts |
| - **The presence of interpreters** - Interpreters orally translated the information brochure and consent form - An additional consent form was signed by interpreters with the statement that (s)he ensured confidentiality and that the woman fully understood the translation of the information brochure and consent form |
| - **Sensitivity to the women's needs & awareness of bias** - Being sensitive to the women's needs of taking a break during the interviews *(e.g. due to the care of children, urgent household tasks)* was crucial - Awareness of possible bias (e.g. risk of missing out, privacy and confidentiality issues) was necessary when other people (family members, friends) entered the interview setting - The interviewer regularly checked for (non-)verbal signs if the women felt uncomfortable or wanted to stop the interview |
